# Are Risk Factors for Postoperative Significant Hemorrhage following Total Knee Arthroplasty Potentially Modifiable? A Retrospective Cohort Study

**DOI:** 10.3390/jpm12030434

**Published:** 2022-03-10

**Authors:** En-Bo Wu, Kuo-Chuan Hung, Sin-Ei Juang, Jo-Chi Chin, Hsiao-Feng Lu, Jih-Yang Ko

**Affiliations:** 1 Department of Anesthesiology, Kaohsiung Chang Gung Memorial Hospital, Chang Gung University College of Medicine, No. 123, Ta-Pei Rd., Niao-Song Dist., Kaohsiung City 833, Taiwan; enbofive@gmail.com (E.-B.W.); juang5251@cgmh.org.tw (S.-E.J.); 2 Department of Anesthesiology, Chi Mei Medical Center, No. 901, Zhonghua Rd., Yongkang Dist., Tainan City 710, Taiwan; ed102605@gmail.com; 3 Department of Anesthesiology, Park One International Hospital, No. 100, Bo’ai 2nd Rd., Zuoying Dist., Kaohsiung City 813, Taiwan; jochi731@gmail.com; 4 Department of Orthopedic Surgery, Kaohsiung Chang Gung Memorial Hospital, Chang Gung University College of Medicine, No. 123, Ta-Pei Rd., Niao-Song Dist., Kaohsiung City 833, Taiwan

**Keywords:** total knee arthroplasty, hemoglobin drop, modifiable risk factors, postoperative hemorrhage, anesthesia

## Abstract

**Simple Summary:**

Total knee arthroplasty is the treatment of choice for end-stage osteoarthritic knees. However, the surgery-associated postoperative hemorrhage remains a big concern. Most of previous studies have defined meaningful postoperative bleeding as blood loss > 500 mL or a hemoglobin (Hb) drop > 20 g/L. The aims of this study were to identify factors that closely related to significant postoperative hemorrhage and whether the identified factors could be modified. Five factors that closely related to more hemorrhage were identified; they were male patients, old age patients, patients without using tranexamic acid (TXA), patients under general anesthesia, and platelet count. Of these five factors, the number of platelets, use of TXA, and spinal anesthesia are perhaps modifiable to limit hemorrhage. These three potentially modifiable factors need to be taken into consideration when making both the preoperative care and anesthesia plan by surgeons and anesthesiologists, especially in male and aged patients.

**Abstract:**

Total knee arthroplasty (TKA) is the treatment of choice for end-stage osteoarthritis (OA) of the knee, because it alleviates pain and restores function of the knee. However, TKA-associated hemorrhage and subsequent anemia remain a concern. Most previous studies have defined meaningful postoperative bleeding as blood loss > 500 mL or hemoglobin (Hb) drop > 20 g/L. Therefore, we defined significant hemorrhage as a postoperative Hb drop more than 20 g/L in this study, and we investigated possible risk factors related to significant hemorrhage in TKA and whether these risk factors are modifiable. This retrospective study was conducted through a comprehensive review of the perioperative records of patients with OA of the knee who underwent TKA between January 2009 and December 2015 at our hospital. Patients were allocated into two groups: patients in Group A had their Hb drop ≤ 20 g/L; patients in Group B had their Hb drop > 20 g/L. Factors analyzed included sex, age, body mass index (BMI), the American Society of Anesthesiologists (ASA) classification, comorbidities, preoperative platelet count, use of tranexamic acid (TXA), operation time, and type of anesthesia. A total of 3350 patients met the criteria for analysis, with 1782 patients allocated to Group A and 1568 patients to Group B. Five independent risk factors for significant hemorrhage were identified: male sex (odds ratio(OR), 1.29; 95% confidence interval(CI), 1.08–1.53; *p* = 0.005), age (OR, 1.02; 95% CI, 1.01–1.03; *p* = 0.001), use of TXA (OR, 0.39; 95% CI, 0.34–0.45; *p* < 0.001), spinal anesthesia versus general anesthesia (OR, 0.71; 95% CI, 0.56–0.90; *p* = 0.004), and preoperative platelet count (OR, 0.96; 95% CI, 0.93–0.98; *p* = 0.001). Of these identified risk factors, preoperative platelet count, use of TXA, and spinal anesthesia are modifiable. These potentially modifiable risk factors need to be taken into consideration when making both the perioperative care and anesthesia plan by surgeons and anesthesiologists, especially in patients at risk of significant hemorrhage.

## 1. Introduction

Osteoarthritis (OA) is strongly associated with aging and is one of the leading causes of physical disability in the elderly population [1]. A rapidly growing demand for total knee arthroplasty (TKA) is anticipated, as life expectancy is increasing in both developed and developing countries. New anatomic designs of prostheses and prostheses made of increasingly durable materials are continuously introduced into the artificial joint market, and the success of the contemporary TKA has increased and has become widely recognized in the recent years.

Despite the above success, TKA-associated hemorrhage remains a major concern [2,3,4,5]. Although several measures, such as preoperative iron supplementation, intraoperative use of tourniquets, hypotensive anesthesia, postoperative drain clamping, or various pharmaceuticals, have been introduced to reduce intraoperative or postoperative hemorrhage in TKA [6,7,8,9], a substantial number of patients still need blood transfusions after TKA [8,10,11]. Postoperative anemia may induce tissue hypoxia due to a reduced oxygen-carrying capacity. The consequences of tissue hypoxia include acute stroke, myocardial ischemia or infarction [12,13], acute kidney injury [14,15], and delirium [16]. A significant postoperative hemorrhage may sometimes require blood transfusion, which is guided by numerous statements and guidelines from professional associations [17,18,19,20,21]. A blood transfusion remains the last resort for the treatment of significant hemorrhage, though patients may face the risk of infections and immunological or transfusion reactions [22,23,24].

Most previous studies have defined meaningful postoperative bleeding as blood loss > 500 mL or a hemoglobin (Hb) drop > 20 g/L [25,26,27,28]. Furthermore, there have been prospective studies [2,5] and retrospective studies [4,11,29] that showed that sex, age, tourniquet time, operation time, preoperative Hb value, and BMI were risk factors related to hemorrhage in TKA. However, many of these risk factors reported in previous studies were not consistently demonstrated as independent predictors for a significant hemorrhage in TKA.

Due to these reasons, this present study used the postoperative Hb drop as an indicator for significant hemorrhage in TKA to assess two insufficiently addressed issues: can independent risk factors of significant hemorrhage in TKA be identified, and are these factors potentially modifiable? We investigated these issues through a comprehensive review of perioperative records of patients who underwent TKA from our single-center database.

## 2. Materials and Methods

The study was approved by the Institutional Review Board of our hospital (IRB number: 202102014B0). All methods were performed in accordance with the relevant guidelines and regulations. Medical, perioperative records of patients who underwent primary TKA due to end stage osteoarthritis, from January 2009 to December 2015, were retrieved from the hospital’s electronic database. Exclusion criteria were patients who received bilateral TKA in a single operation; computer-assisted navigation surgery; patients with medial calcification of the femoral artery and preoperative anemia; patients with rheumatoid arthritis, liver cirrhosis, and end stage renal disease; and incomplete chart records.

### 2.1. Definition of Significant Hemorrhage

We defined a hemoglobin (Hb) drop as the difference between the preoperative Hb value and the lowest recorded postoperative Hb value, which was recorded at the third postoperative day as in previous studies [30,31] on TKA. As in previous studies [25,26,27,28], we divided our patients into two groups according to the Hb drop. We classified a Hb drop ≤ 20 g/L as the control group (Group A), while a Hb drop > 20 g/L was defined as the significant hemorrhage group (Group B).

### 2.2. Choice of Anesthesia

The surgeries were performed under general anesthesia or spinal anesthesia. If the patient failed to meet the guidelines for the usage of antiplatelet or anticoagulant agents, general anesthesia would be performed instead of spinal anesthesia. In addition, the general contraindications of spinal anesthesia were also considered in our patients, especially in those who refused spinal anesthesia.

### 2.3. The Management of Perioperative Bleeding

In the routine practice of our center, patients are treated with Hb < 100 g/L [32], moderate-to-severe thrombocytopenia (platelet count < 10 × 10^4^/μL) [33], and an INR/aPTT ratio < 1.50 [34] before the noncardiac surgery. However, in our study, none of the enrolled patients had impaired hemostasis. As a standard practice in our hospital, the intraoperative use of tourniquets is routine in every patient undergoing TKA, and inflation pressures were set at 250 to 300 mmHg. All patients treated with antiplatelet or anticoagulant agents were asked to withhold these medications for one week or days, which were followed by the guidelines prior to surgery [35,36]. The use of tranexamic acid (TXA) depended on whether the patient was at a high risk of bleeding, as judged by orthopedic surgeons, such as in diabetes, chronic kidney disease, preoperative anemia, and the use of antiplatelet or anticoagulant drugs.

The following information was included for analysis: sex, age, ASA physical status, body mass index (BMI), comorbidities, preoperative platelet count, type of anesthesia (general/spinal), operation time (general/spinal), blood pressure, intraoperative fluid supply, and use of TXA. For the intraoperative intravenous fluid supply, patients were basically supplied with a lactate ringer solution with a flow rate of 2–3 mL/kg/h; however, infusion rates could be adjusted in different clinical conditions by anesthesiologists. The outcome variables of this study were independent risk factors of a significant Hb drop in TKA patients.

## 3. Statistical Analysis

Continuous numeric variables were tested by the Kolmogorov–Smirnov test for normal distribution. A student’s *t*-test was used to test normally distributed data. Non-normally distributed data were compared using the Mann–Whitney U test and expressed as the median (interquartile range, IQR). The chi-square or Fisher’s exact test was used to analyze categorical variables. A univariate analysis and multiple logistic regression model were used to determine the influence of each variable on the Hb drop. Data are presented as the raw numbers or percentages. Numerical data are presented as the median (25–75%). Statistical significance was set at *p* < 0.05.

## 4. Results

**Figure 1:**
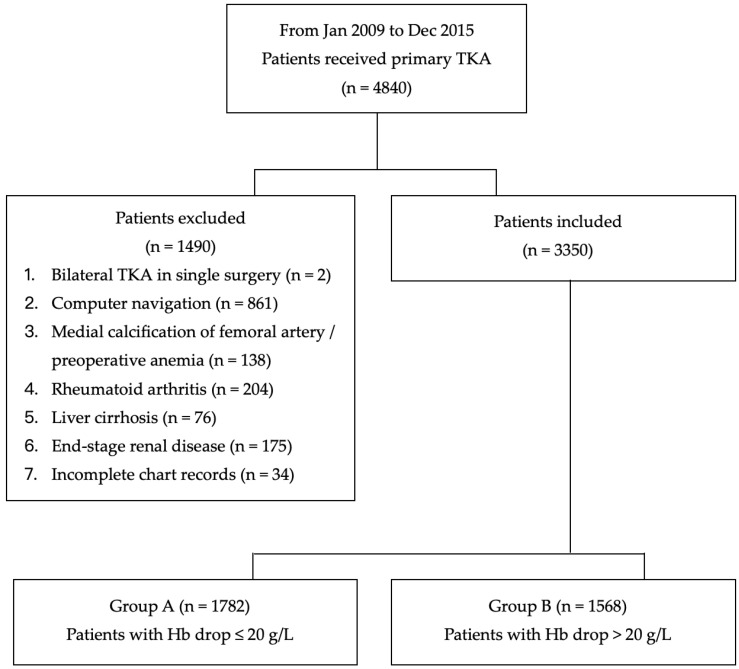
Flow chart of the study. TKA, total knee arthroplasty; Hb, hemoglobin.

A total of 4840 perioperative records of patients who underwent primary TKA for an OA knee were retrieved from the hospital’s database (Figure 1). After excluding patients who received bilateral TKA in a single surgery (*n* = 2); received computer-assisted navigation surgery (*n* = 861); had medial calcification of the femoral artery and preoperative anemia (*n* = 138); had rheumatoid arthritis (*n* = 204), liver cirrhosis (*n* = 76), and end stage renal disease (*n* = 175); and patients with incomplete chart records (*n* = 34), a total of 3350 patients were included in the study. There were 1782 patients in Group A and 1568 patients in Group B.

Table 1 summarizes the demographics of the patients in Group A and B. The proportion of male patients in Group B was higher than male patients in Group A (25.8% vs. 19.4%, *p* < 0.001). The median age of patients in Group B was higher than patients in Group A (71.0 years vs. 69.0 years, *p* < 0.001). A difference in the distribution of the ASA physical status was noted. Group B had a higher proportion of patients with ASA 3 than Group A (35.0% vs. 39.6%, *p* = 0.019). More patients in Group B had ischemic heart disease than in Group A (7.8% vs. 4.7%, *p* < 0.001). The proportion of patients who received TXA treatment in Group A was higher than that in Group B (54.4% vs. 31.6%, *p* < 0.001). The median preoperative Hb values were similar between patients in Group A and Group B (131 g/L vs. 132 g/L, *p* = 0.193). The median postoperative Hb value of patients in Group A was higher than that of patients in Group B (115 g/L vs. 106 g/L, *p* < 0.001). Patients in Group B had a lower platelet count than patients in Group A (22.2 × 10^4^/μL vs. 22.9 × 10^4^/μL, *p* < 0.001). The prothrombin time (PT) or activated partial thromboplastin time (aPTT) was similar in Group A and B. The operation time (general/spinal), type of anesthesia, BMI, Hb value at discharge, and prevalence of diabetes mellitus and hypertension were similar in the two groups.

For quantitative statistical analyses, univariate and multiple logistic regression analyses were performed to explore any independent predictors for significant hemorrhage (Table 2). Male sex (odds ratio OR], 1.29; 95% confidence interval [CI], 1.08–1.53; *p* = 0.005) was an independent predictor for significant hemorrhage, i.e., the risk of an Hb drop > 20 g/L in males was 1.29 times higher than in females. Age (OR 1.02; 95% CI, 1.01–1.03; *p* = 0.001) was an independent predictor for significant hemorrhage, i.e., for each one year of increasing age, the risk of an Hb drop > 20 g/L was 1.02 times higher. The use of TXA (OR, 0.39; 95% CI, 0.34–0.45; *p* < 0.001) was an independent predictor for significant hemorrhage i.e., when TXA was used, the risk of an Hb drop > 20 g/L was 0.39 times lower. Spinal anesthesia (OR, 0.71; 95% CI, 0.56–0.90; *p* = 0.004) was an independent predictor for significant hemorrhage, i.e., in patients who had spinal anesthesia, the risk of an Hb drop > 20 g/L was 0.71 times lower compared with patients who had general anesthesia. Platelet count (OR, 0.96; 95% CI, 0.93–0.98; *p* = 0.001) was an independent risk factor for significant hemorrhage, i.e., for every 2 × 10^4^/μL increase in platelet count, the risk of an Hb drop > 20 g/L was 0.96 times lower. The ASA physical status, BMI, ischemic heart disease, diabetes mellitus, hypertension, operation time, PT, and aPTT were not identified as independent risk factors in our univariate or multivariate logistic regression model. Fifty-eight patients in Group B received a blood transfusion postoperatively for postoperative Hb values that were below 80 g/L. No patients in Group A received a blood transfusion.

## 5. Discussion

Intraoperative and postoperative hemorrhage is inevitable in TKA because TKA involves extensive bone resection [37] and soft tissue manipulation [38,39], and often, this hemorrhage is substantial [40]. Intraoperative hemorrhage in TKA might be reduced because a tourniquet is applied during the operation [41]; however, significant hemorrhage may occur postoperatively after the tourniquet is removed [42]; usually, the lowest Hb level was recorded at the third postoperative day. Medial calcification of the femoral artery is an important cause for a significant hemorrhage in TKA [43,44,45], and intraoperative blood transfusion should be considered in these patients. Two main reasons for blood transfusions in the operating room or at a post-anesthesia care unit in our study were patients with medial calcification of the femoral artery (typical ‘railroad trucks’ pattern in the scanogram of the lower extremity) and patients with preoperative anemia. We excluded these patients to avoid the effects of arterial calcification or anemia of an undetermined etiology on postoperative bleeding. The total hemorrhage in TKA is usually counted as the sum of the intraoperative hemorrhage and postoperative drainage. However, this may underestimate the true loss, as it ignores the so-called “hidden” loss [46], which includes extravasation of blood into the knee and surrounding tissues and hemolysis, hence why we used Hb drop as an estimate of the true total hemorrhage in TKA. The total number of patients who required a blood transfusion was 58. We chose not to define a significant hemorrhage by the requirement for a blood transfusion alone, as the absolute number and percentage of patients who received a postoperative transfusion was so low that results may not have been reliable. Instead, we chose to define patients with a significant hemorrhage based on the mean Hb drop to form two groups, as this would have given us the most power for analysis. There was a noticeable difference between the two groups’ mean Hb drop, with Group A at 1.35 ± 0.50 SD and Group B at 2.80 ± 0.61 SD. This supported our definition of a significant hemorrhage as a valid but also powerful classification for analysis. All patients who received a blood transfusion were in the significant hemorrhage group.

Males had a higher Hb drop than females in this study, and this finding was in keeping with previous studies [3,5,29,47]. Our results showed that the risk of a significant hemorrhage in males was 1.29 time higher than in females. Group B, with a higher proportion of males, had a slightly higher pre-operative Hb than Group A. This may have been due to the inherent difference in the baseline Hb levels between male and females; women were shown to have mean Hb levels approximately 12% lower than men [48]. One may argue that since females have a lower blood volume and lower Hb value as compared with males, females would tend to have a greater impact on the postoperative Hb drop; however, this argument assumes that females and males are the same in every aspect. A retrospective cohort study conducted by Drago et al. observed that about 2 weeks after the surgery, the level of Hb recovery in males was significantly higher than in females [49]. They explained that the reason may be associated to the male anatomy, body mass index, and characteristics. The impact of the sex difference and sex hormonal level on the perioperative hemorrhage will be another interesting topic for future studies. The best cut-off value of reduced hemoglobin for a significant hemorrhage was 20 g/L, irrespective of the gender [24,25].

Increasing age was related to a significant hemorrhage. Patients in Group B were older than patients in Group A (Table 1). Previous studies [10,50,51] have also shown that increasing age is associated with a higher hemorrhage in TKA, and it is postulated that muscle wasting, arterial stiffness due to medial calcification, arterial hypertension, and inadequate postoperative pain treatment in elderly patients are the causes for a higher postoperative hemorrhage. Furthermore, our results showed that with every one-year age increase, the risk of a significant hemorrhage was 1.02 times higher. Another interesting finding was that the platelet count was lower in Group B, while their mean age was higher. The accepted definition of perioperative thrombocytopenia is a platelet count less than 15 × 10^4^/μL [52]. While no patients were thrombocytopenic in this study, our results showed that for every 2 × 10^4^/μL increase in the preoperative platelet count, the risk of significant hemorrhage was 0.96 times lower. It is reasonable to speculate that ceiling effects of platelets were reached in these elderly patients. In addition to platelet count, the inherent function of platelets is also crucial to the formation of blood clots. Aging is associated with a lower hematopoietic regenerative capacity, and this may explain why those inherent functions of platelets are lower in the elderly population [53,54].

It is widely accepted that TXA is safe and effective in reducing the total hemorrhage in TKA [55,56]. More patients in Group A had TXA treatment (Table 1), and the beneficial effect of TXA was further exemplified in the univariate and multivariate logistic regression model (Table 2). The risk of a significant hemorrhage in patients having TXA was 0.39 times lower as compared with patients without TXA. Although there are limited data on the safety of TXA, current clinical practice guidelines still suggest that TXA does not increase venous or arterial thromboembolic events, even if the patient has a relevant past medical history [57].

There have been many studies discussing the effects of general anesthesia and spinal anesthesia on the hemorrhage in TKA. An early systematic review of 141 trials, including 9559 patients, showed that the neuraxial blockade reduced the odds of requiring a blood transfusion by 50%; however, this review was not specific for TKA and included a range of non-orthopedic surgeries [58]. Two early systematic reviews did not identify sufficient evidence that the anesthesia method influenced hemorrhaging in TKA [59,60]. On the other hand, three recent studies showed that patients that received spinal anesthesia in TKA required fewer transfusions [61,62,63]. Juelsgaard et al. noted that hypotension caused by neuraxial anesthesia has been shown to reduce surgical hemorrhage in TKA [64]. Spinal anesthesia leads to sympathectomy, which results in a decreased vascular smooth muscle tone, including a decreased systemic vascular resistance and decreased venous return. These effects, combined with the subsequent reduction in cardiac output, ultimately help control the surgical bleeding [65]. The beneficial effect of spinal anesthesia in reducing hemorrhage was exemplified in our logistic regression model. The risk of significant hemorrhage in patients who received spinal anesthesia was 0.71-times lower than patients who received general anesthesia.

There are limitations to our study. Our study may suffer from a potential bias inherent to retrospective studies. The risk factors for a postoperative significant hemorrhage following TKA that we analyzed were only limited to retrospective data, and we could not predict whether they would be confirmed in a prospective evaluation. More and more studies, including randomized controlled studies [66], meta-analysis studies [67,68], and systematic reviews of the Cochrane database [69], have shown no difference in blood transfusions with or without tourniquets for TKA. However, in our department of orthopedics, tourniquets are routinely used for TKA. The reason is that intraoperative hemorrhage may be reduced, which allows for a clearer definition of the surgical field. Therefore, our results cannot be concluded from TKA without tourniquets. The difference in the surgical experience among orthopedic surgeons was not considered in this study. In our center, TKA is performed by only three orthopedic surgeons, each with over 20 years of experience. However, we still believe in the existence of surgical bias. The types of surgical approach performed in the study, namely the midline approach, the para-patella approach, and the mid sub-vastus approach, were not analyzed; in fact, all the surgical procedures were performed by senior orthopedic surgeons in this study. Computer-assisted navigation was excluded in this study, because this relatively novel device was first introduced at the beginning of the study period, and hemorrhage may have varied between different orthopedic surgeons and their varying level of experience with this device. Even Xie et al. demonstrated that there was no significant difference in the total surgical blood loss and other outcomes between computer-assisted navigation and conventional TKA at a significantly longer operation time in their meta-analysis of 21 randomized controlled trials [70]. Our cut-off point for a significant hemorrhage point, a Hb drop ≤ 20 g/L, may not apply to other institutions and other populations of patients. Finally, our results suggested that for every preoperative 2 × 10^4^/μL platelet increase, it would decrease the risk of hemorrhage by 0.96. However, preoperative platelet transfusion is never encouraged for controlling a hemorrhage in any surgery, except for those patients with preoperative thrombocytopenia. Although the beneficial effect of “every preoperative 2 × 10^4^/μL platelet increase” is only a mathematical deduction, it brings out an interesting question of if there are any ceiling effects of platelet counts or any subtle effects of platelets that we have ignored.

## 6. Conclusions

Through our retrospective study, we found that sex and age were non-modifiable risk factors for a significant hemorrhage, while the preoperative platelet count, the use of TXA, and the type of anesthesia were potentially modifiable factors. Further prospective studies are required to verify our results, especially the role of platelet transfusions in elderly patients and the type of anesthesia. We recommend that TXA should be given routinely at TKA to reduce the risk of significant hemorrhage; this evidence is also supported by other studies. Finally, our mathematical model for the “platelet count increase” would never be our recommendation for a preoperative platelet transfusion before further experimental studies and stringent examinations.

## Figures and Tables

**Table 1:** Demographic and characteristic features of patients undergoing total knee arthroplasty.

Features	*n* (%)/Median (IQR)	Group A	Group B	*p* Value
Sex				
Female	2601 (77.6%)	1437 (80.6%)	1164 (74.2%)	<0.001
Male	749 (22.4%)	345 (19.4%)	404 (25.8%)	
Age	70.0 (64.0–75.0)	69. 0(64.0–74.0)	71.0 (65.0–76.0)	<0.001
ASA				
Ⅰ	63 (1.9%)	37 (2.1%)	2 6(1.7%)	0.019
Ⅱ	2042 (61.0%)	1121 (62.9%)	921 (58.7%)	
Ⅲ	1245 (37.2%)	624 (35.0%)	621 (39.6%)	
BMI				
BMI < 18.5	8 (0.2%)	3 (0.2%)	5 (0.3%)	0.372
BMI = 18.5–24	565 (16.9%)	289 (16.2%)	276 (17.6%)	
BMI > 24	2777 (82.9%)	1490 (83.6%)	1287 (82.1%)	
Ischemic heart disease				
No	3143 (93.8%)	1698 (95.3%)	1445 (92.2%)	<0.001
Yes	207 (6.2%)	84 (4.7%)	123 (7.8%)	
Diabetes Mellitus				
No	2557 (76.3%)	1368 (76.8%)	1189 (75.8%)	0.524
Yes	793 (23.7%)	414 (23.2%)	379 (24.2%)	
Hypertension				
No	1217 (36.3%)	655 (36.8%)	562 (35.8%)	0.583
Yes	2133 (63.7%)	1127 (63.2%)	1006 (64.2%)	
Tranexamic acid				
No	1885 (56.3%)	813 (45.6%)	1072 (68.4%)	<0.001
Yes	1465 (43.7%)	969 (54.4%)	496 (31.6%)	
Operation time (h)				
<2 h	345 (10.3%)	194 (10.9%)	151 (9.6%)	0.232
>2 h	3005 (89.7%)	1588 (89.1%)	1417 (90.4%)	
Anesthesia				
General	3002 (89.6%)	1580 (88.7%)	1422 (90.7%)	0.055
Spinal	348 (10.4%)	202 (11.3%)	146 (9.3%)	
Preoperative Hb (g/L)	133 (124–141)	131 (123–139)	132 (122–142)	0.193
Lactate Ringer fluid (ml)	408.2 (358.8–460.4)	403.5 (360.2–457.7)	410.8 (356.2–465.4)	0.396
Postoperative Hb (g/L)	111 (102–119)	115 (107–122)	106 (97–115)	<0.001
Platelet count (10^4^/μL)	22.7 (1.92–2.65)	22.9 (1.95–2.69)	22.2 (1.88–2.61)	<0.001
PT (s)	10.1 (9.9–10.4)	10.1 (9.9–10.4)	10.1 (9.9–10.4)	0.765
aPTT (s)	27.3 (26.0–28.6)	27.3 (26.0–28.5)	27.3 (26.0–28.7)	0.478
Discharge Hb (g/L)	123 (115–131)	123 (115–131)	124 (114–132)	0.215

PT: prothrombin time, aPTT: activated partial thromboplastin time.

**Table 2:** Univariate and multiple logistic regression model of the postoperative Hb drop.

Variables (Unit)	*n* (%)/Unit	Uariate		Multivariate	
		OR (95% CI)	*p* Value	OR (95% CI)	*p* Value
Sex					
Female	2601 (77.6%)	1		1	
Male	749 (22.4%)	1.45 (1.23–1.70)	<0.001	1.29 (1.08–1.53)	0.005
Age	70 (64–75)	1.02 (1.01–1.03)	<0.001	1.02 (1.01–1.03)	0.001
ASA					
Ⅰ	63 (1.9%)	1		1	
Ⅱ	2042 (61.0%)	1.17 (0.70–1.95)	0.547	1.07 (0.63–1.82)	0.793
Ⅲ	1245 (37.2%)	1.42 (0.75–2.37)	0.184	1.09 (0.64–1.86)	0.758
BMI					
BMI < 18.5	8 (0.2%)	1		1	
BMI = 18.5–24	565 (16.9%)	0.57 (0.14–2.42)	0.449	0.56 (0.12–2.50)	0.444
BMI > 24	2777 (82.9%)	0.52 (0.12–2.17)	0.369	0.51 (0.11–2.28)	0.377
Ischemic heart disease					
No	3143 (93.8%)	1		1	
Yes	207 (6.2%)	1.72 (1.29–2.29)	<0.001	1.19 (0.88–1.62)	0.262
Diabetes Mellitus					
No	2557 (76.3%)	1		1	
Yes	793 (23.7%)	1.05 (0.90–1.24)	0.524	1.04 (0.88–1.24)	0.625
Hypertension					
No	1217 (36.3%)	1		1	
Yes	2133 (63.7%)	1.04 (0.90–1.20)	0.583	1.00 (0.86–1.17)	0.976
Tranexamic acid					
No	1885 (56.3%)	1		1	
Yes	1465 (43.7%)	0.39 (0.34–0.45)	<0.001	0.39 (0.34–0.45)	<0.001
Operation time (h)					
<2 h	345 (10.3%)	1		1	
>2 h	3005 (89.7%)	1.15 (0.92–1.44)	0.233	0.95 (0.75–1.21)	0.685
Anesthesia					
General	3002 (89.6%)	1		1	
Spinal	348 (10.4%)	0.80 (0.64–1.01)	0.056	0.71 (0.56–0.90)	0.004
Platelet count ^#^ (2 × 10^4^/μL)	0.45 (0.38–0.53)	0.95 (0.93–0.97)	<0.001	0.96 (0.93–0.98)	0.001
PT (s)	10.1 (9.9–10.4)	1.07 (0.95–1.20)	0.289	0.98 (0.86–1.11)	0.745
aPTT (s)	27.3 (26.0–28.6)	1.01 (0.98–1.04)	0.441	1.01 (0.98–1.04)	0.550

PT: Prothrombin time, aPTT: activated partial thromboplastin time, ^#^ one unit of platelet count is 2 × 10^4^/μL.

## Data Availability

The data presented in this study are available from the corresponding authors upon reasonable request.
